# Supplementary figures and images for: Single-Cell Transcriptome Analysis Reveals RGS1 as a New Marker and Promoting Factor for T-Cell Exhaustion in Multiple Cancers
Source: Front Immunol. 2021 Dec 8;12:767070. doi: 10.3389/fimmu.2021.767070 (PMC8692249; doi:10.3389/fimmu.2021.767070)

A

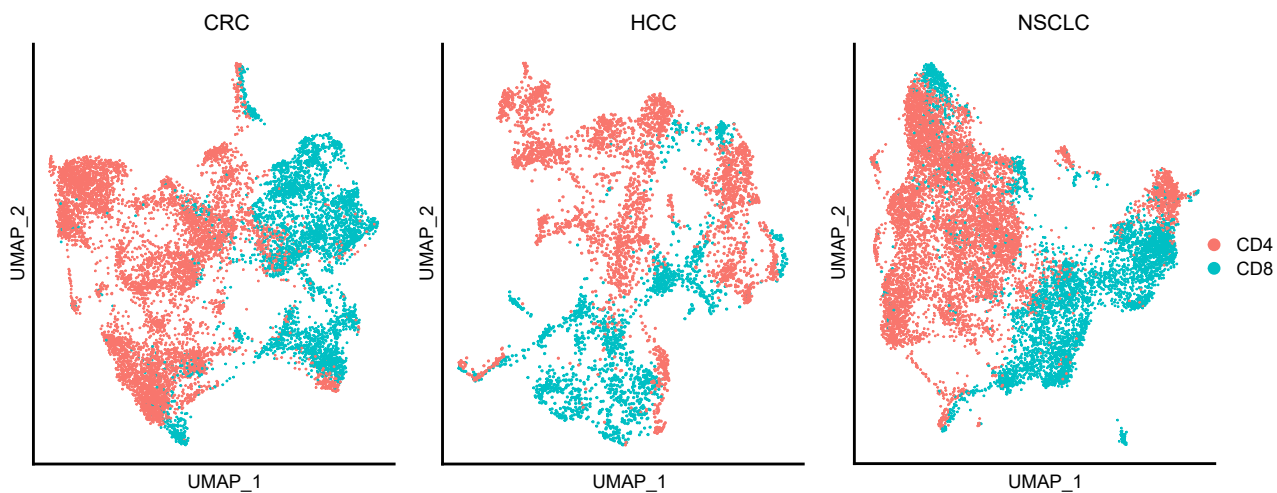

B

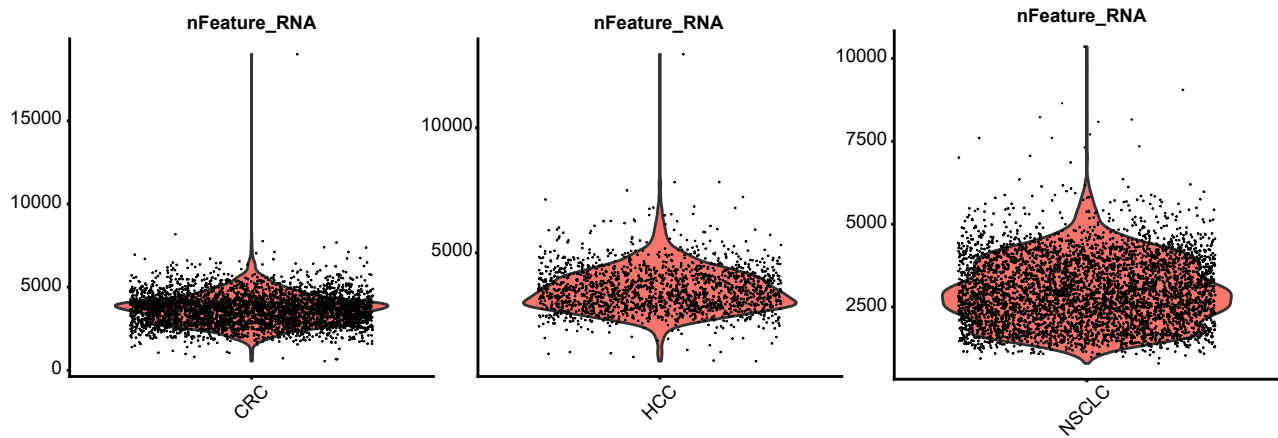

C

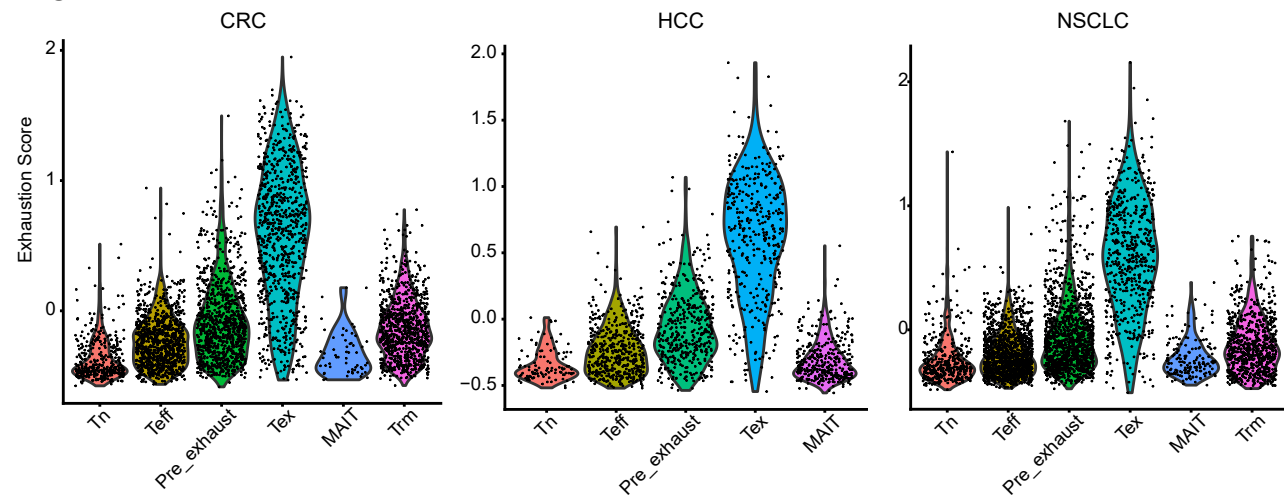

Supplement: Supplementary Figure 1 — Clustering of CD8+ T cells in three cancers. A. UMAP visualization of T cells in three cancers. B. The number of detected genes in each cell before filter in three cancers. C. The exhaustion scores of different cell types using the exhaustion gene list including HAVCR2, TIGIT, LAG3, PDCD1, CXCL13, LAYN, TOX, CTLA4, BTLA. [file Image_1.pdf]

CRC

HCC

NSCLC

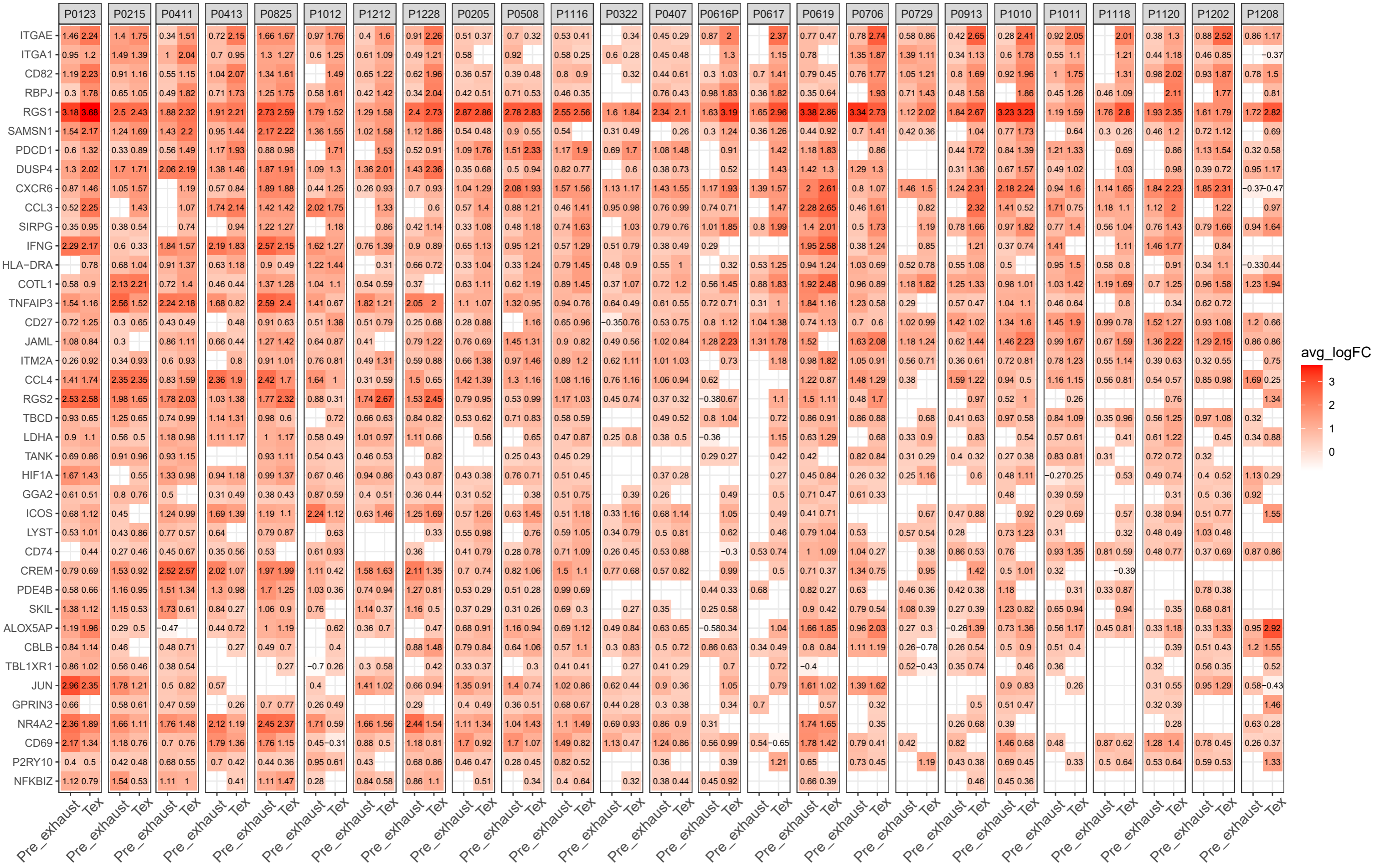

Supplement: Supplementary Figure 2 — The correlation coefficient between RGS1 and Candidate gene set of Tex cells in CD8+ T cells of different patients. [file Image_2.pdf]

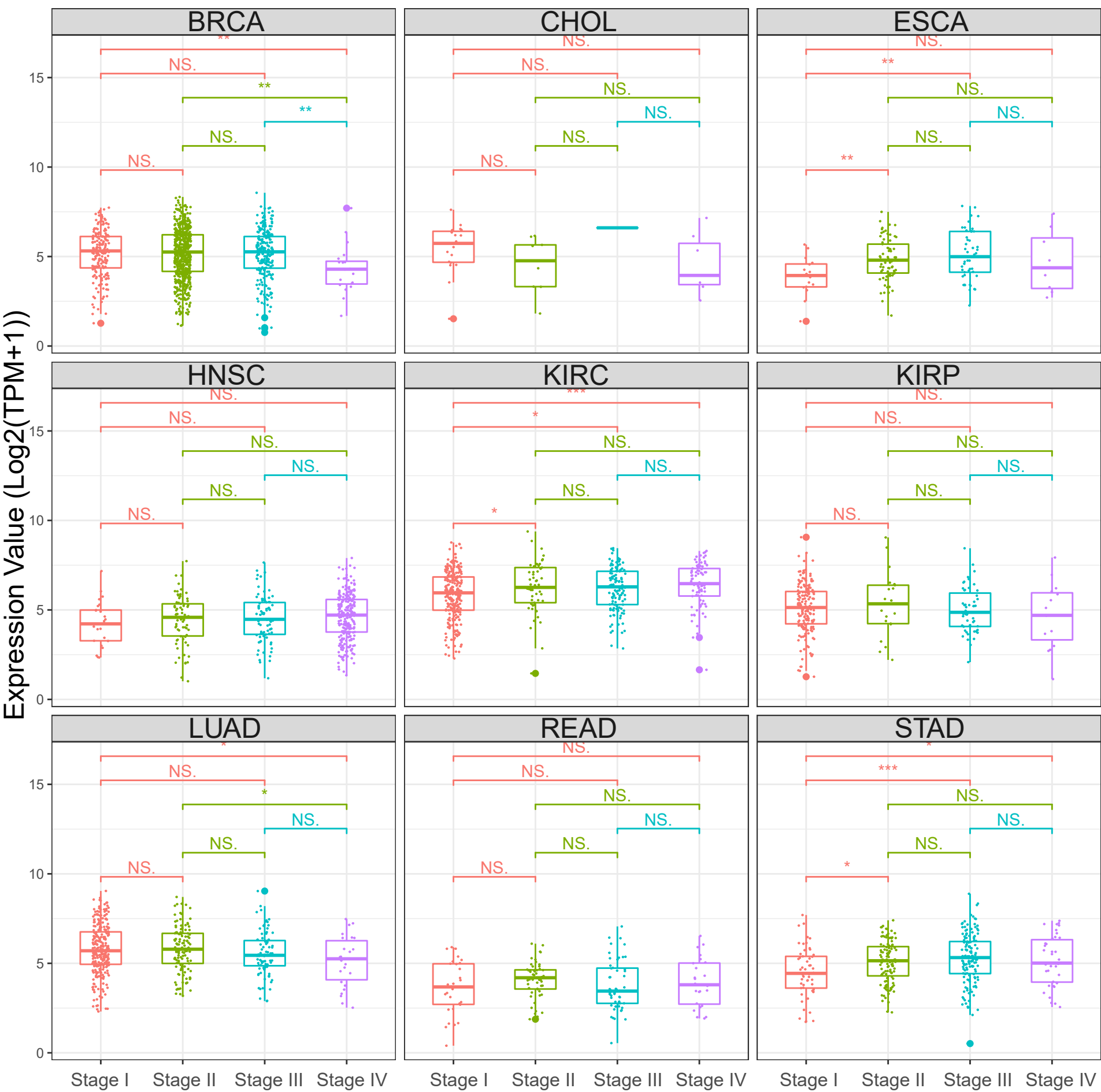

Supplement: Supplementary Figure 3 — The mRNA expression value of RGS1 of different stages in TCGA database. [file Image_3.pdf]
